# Exploring the causal relationship between immune cells and idiopathic pulmonary fibrosis: a bi-directional Mendelian randomization study

**DOI:** 10.1186/s12890-024-02942-w

**Published:** 2024-03-20

**Authors:** Zhao He, Ruixin Wang, Chenghu Song, Jiwei Liu, Ruo Chen, Mingfeng Zheng, Weici Liu, Guanyu Jiang, Wenjun Mao

**Affiliations:** Department of Thoracic Surgery, The Affiliated Wuxi People’s Hospital of Nanjing Medical University, Wuxi People’s Hospital, Wuxi Medical Center, Nanjing Medical University, No. 299 Qingyang Rd, Wuxi, 214023 China

**Keywords:** Idiopathic pulmonary fibrosis, Immunophenotype, Immune cell, Mendelian randomization, Sensitivity analysis

## Abstract

**Background:**

The potential pathogenic mechanism of idiopathic pulmonary fibrosis is widely recognized to involve immune dysregulation. However, the current pool of studies has yet to establish a unanimous agreement regarding the correlation between various types of immune cells and IPF.

**Methods:**

By conducting a two-sample Mendelian randomization analysis using publicly available genetic data, the study examined the causal relationship between IPF and 731 immune cells. To ensure the reliability of the results, combined sensitivity analyses and inverse Mendelian analyses were conducted. Moreover, within subgroups, multivariate Mendelian randomization analyses were utilized to investigate the autonomous causal connection between immune cell characteristics and IPF.

**Results:**

After adjusting for false discovery rate, it was discovered that 20 immunophenotypes exhibited a significant association with IPF. After subgrouping for multivariate Mendelian randomization analysis, there were six immunophenotypes that remained significantly associated with IPF. These included CD33 + HLA DR + CD14dim (OR = 0.96, 95% CI 0.93–0.99, *P* = 0.033), HLA DR + NK (OR = 0.92, 95% CI 0.85–0.98, *P* = 0.017), CD39 + CD8 + T cell %T cell (OR = 0.93, 95% CI 0.88–0.99, *P* = 0.024), CD3 on activated & secreting Treg (OR = 0.91, 95% CI 0.84–0.98, *P* = 0.026), PDL-1 on CD14- CD16 + monocyte (OR = 0.89, 95% CI 0.84–0.95, *P* = 8 × 10^–4^), and CD45 on CD33 + HLA DR + CD14- (OR = 1.08, 95% CI 1.01–1.15, *P* = 0.011).

**Conclusion:**

Our study reveals a noteworthy association between IPF and various immune cells, providing valuable insights for clinical research and aiding the advancement of immunologically-based therapeutic strategies.

**Supplementary Information:**

The online version contains supplementary material available at 10.1186/s12890-024-02942-w.

## Introduction

The incidence of idiopathic pulmonary fibrosis (IPF) is increasing annually, and it is classified as a chronic and fatal interstitial lung disease [1]. According to statistics, it constitutes approximately two-thirds of the diagnosed population aged 60 and above, with a higher prevalence in men compared to women. It affects 10–20 individuals per 100,000 in Europe and the United States, and patients with IPF typically live less than three years on average [2, 3]. However, complete treatment of IPF can only be achieved through lung transplantation techniques. Currently, the main clinical applications to slow down the progression of IPF are FDA-approved nidazanib and pirfenidone. The complexity and unknown etiology of IPF are the primary reasons for its challenging nature [4–6].

The cause of IPF is still not fully understood, although research has indicated that inflammation and immune dysregulation play a role. However, the specific immune-related mechanisms are not well understood and are a subject of controversy [7]. In terms of pathophysiology, immune dysregulation acts as a driving force, and dysregulated wound healing resulting from an immune-inflammatory response is one of the factors contributing to IPF. Furthermore, the presence of elevated pro-fibrotic cytokines (i.e. interleukin-8 (IL-8) and TNF-α) and immune abnormalities in the lungs of patients with IPF support the inflammatory hypothesis [8, 9]. However, attempts to treat IPF by modulating inflammation in clinical trials have unfortunately had negative effects [10–12]. There is growing evidence suggesting that IPF is closely linked to both the innate and adaptive immune systems [13]. Immune cells, including monocytes, T cells, macrophages, and natural killer (NK) cells, are primarily responsible for causing IPF [14]. Cytokines such as IL-6 and IL-23 induce various type 17 immune cells (such as regulatory T cells (Tregs), CD8 + T cells, and NKT cells) to produce IL-17A, which in turn induces TGF-β and leads to pulmonary fibrosis [15]. Targeted therapy for type 17 immunity is considered a new treatment strategy. Therefore, it is expected that more targets that inhibit the development of IPF in an immunological sense will be discovered.

**Fig. 1:**
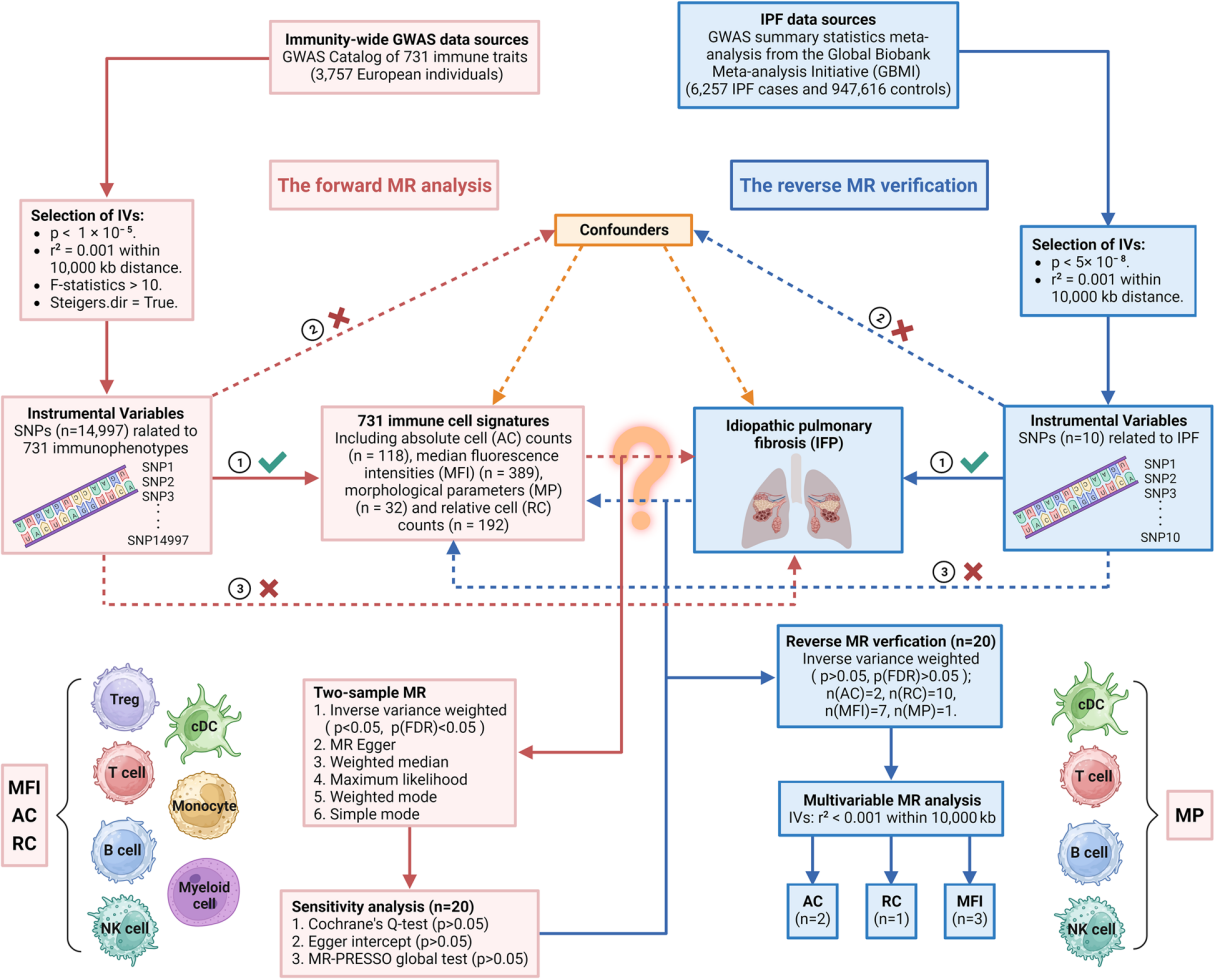
Overall workflow of our research. The Global Biobank Meta-analysis Initiative (GBMI) refers to Genome-wide Association Studies (GWAS). Created with BioRender.com

Mendelian randomization (MR), a crucial epidemiological approach, relies on the independent distribution laws of Mendel. It utilizes SNPs (single nucleotide polymorphisms) to mimic and determine if risk factors impact health outcomes, effectively removing biases from confounding factors and reverse causation. The progress made in genome-wide association studies (GWAS) and MR has facilitated the easier assessment of causal connections between diseases and immune traits [16]. Numerous studies have provided evidence supporting the notion that IPF is linked to immune cells [14, 15]. Hence, we employed extensive two-sample MR analyses to examine the causal relationship between IPF and 731 immune traits. The validity of the findings was reinforced through sensitivity analyses, reverse MR, and multivariate mendelian randomization (MVMR) analyses. The objective of our research is to uncover the potential impact of immune cell characteristics in IPF and potentially identify novel approaches for disease prediction and treatment. Figure 1 depicts the entire research process.

## Materials and methods

### Study design

Analyzing GWAS data, we investigated the reciprocal causal relationships between IPF risk and immune cells (731 samples from seven immune panels). For causal inference to be effective, risk factors and reliable instrumental variables (IVs) must be captured. It is imperative to satisfy three fundamental assumptions: (1) the existence of a direct association between genetic variation and exposure; (2) the absence of correlation between genetic variation and potential confounding factors that lie between exposure and outcome; and (3) the non-influence of genetic variation on the outcome through alternative pathways apart from the exposure. The institutional review boards have approved all studies included in the data.

### Data sources

Summary statistics for immune traits obtained from genome-wide association studies (GWAS) can be accessed through the GWAS Catalog, specifically the catalog entries GCST0001391 to GCST0002121 [16]. The study examined a total of 118 absolute cell (AC) quantities, 389 median fluorescence intensities (MFI) representing surface antigen levels, 192 relative cell quantities (RC), and 32 structural features (MP) using flow cytometry. To analyze the GWAS data, we investigated the reciprocal causal relationships between a specific set of immune cells (across seven immune panels, totaling 731 samples) and the risk of developing diseases. In a population from Sardinia consisting of 3,757 individuals, we evaluated a total of 731 immunotypes. We genotyped the samples using four Illumina arrays (ExomeChip, Cardio-MetaboChip, ImmunoChip, and OmniExpress) and then imputed the genotypes across the entire genome using a reference panel comprised of 3,514 Sardinian sequences. After accounting for covariates such as age, gender, and age squared, we tested the associations using the Sardinian sequence-based reference panel to impute approximately 22 million SNPs genotyped with high-density arrays [17]. As for IPF, the data consisted of 6,257 cases and 947,616 controls, which were obtained from the most recent meta-analysis of GWAS summary statistics conducted by the Global Biobank Meta-analysis Initiative (GBMI) [18].

### Selection of instrumental variables

In accordance with recent research, the significance level of IVs for each immune trait was set to 1 × 10^–5^ [16, 19]. To remove these SNPs (with a linkage disequilibrium [LD] r^2^ threshold < 0.001 within a distance of 10,000 kb) [20], the PLINK software (version v1.90) employed the clumping procedure, with LD r^2^ calculated using the 1000 Genomes Projects as a reference panel. In order to assess the strength of the extracted IVs, we computed the R2 and *F*-value for each IV to prevent any potential instrumental bias caused by weak instruments. We eliminated IVs from our analysis that had low F statistics (*F* < 10). Additionally, to avoid reverse causality and detect whether the correlation between SNPs and outcome was greater than exposure, we conducted the Steiger Test for further screening. For further reverse MR validation, for IPF, we modified the significance level to 5 × 10^ −8^ and identified ten IVs.

### Mendelian randomization

The study utilized various methods, such as inverse variance weighted (IVW), MR-Egger, weighted median, weighted mode, maximum likelihood (ML) and Simple mode, to investigate if there existed a causal link between 731 immunophenotypes and IPF. To obtain an overall estimate of the impact of immunotypes on IPF, the IVW technique employed a meta-analysis approach in conjunction with Wald estimates for each SNP. In the absence of horizontal pleiotropy, the IVW findings would remain unbiased [21]. The MR-Egger method relies on the assumption of instrument strength that is not influenced by direct effects (InSIDE). This assumption allows for the assessment of pleiotropy by considering the intercept term. When the intercept term is zero, it suggests the absence of horizontal pleiotropy, and the outcome of the MR-Egger regression aligns with IVW [22]. The weighted median provides a precise estimation by assuming that a minimum of 50% of the IVs are valid [23]. The ML approach is comparable to IVW, under the assumption that there is no heterogeneity or horizontal pleiotropy. Assuming the hypotheses are met, the outcomes will be impartial, and the standard deviations will be less than IVW [24]. When the InSIDE hypothesis is not upheld, the weighted model estimate has demonstrated enhanced ability in identifying a causal effect, reduced bias, and decreased occurrence of type I errors compared to MR-Egger regression [23]. Despite not being as powerful as IVW, simple mode offers robustness for pleiotropy [25]. In order to evaluate the causal relationship between immunophenotypes and IPF, we conducted reverse MR analysis on the immunophenotypes that were determined to have a causal association with IPF in the forward MR analysis. The techniques and configurations utilized were in line with those of forward MR.

### Sensitivity analysis

The Cochran’s IVW Q statistics were used to assess the diversity of IVs. In case there was diversity in the estimated impact, we substituted the standard fixed-effects IVW with the random-effects IVW technique [26]. To detect the existence of horizontal pleiotropy, the MR-Egger method was employed with caution. If the MR-Egger intercept was found to be statistically significant, it indicated that the association findings might be affected by horizontal pleiotropic effects of other characteristics [27]. To exclude possible horizontal pleiotropy, the global test MR-PRESSO (MR pleiotropy residual sum and outlier) was also performed to determine if there were any outliers [28]. Additionally, as part of "leave-one-out" analysis, each instrumental SNP was omitted in turn in an effort to identify potentially heterogeneous SNPs. The scatter diagrams indicated that the outcomes were not influenced by anomalies, while the Leave-one-out plots exhibited the resilience of the association.

### Multivariable MR

In order to evaluate the independent causal impact of immune cell traits on IPF, we performed the MVMR analysis [29] by categorizing them into various subgroups according to traits type. This expands upon univariate MR by enabling the simultaneous identification of causal effects from multiple risk factors. MVMR considers the interrelation between different immune phenotypes within subgroups [30]. The SNPs utilized for conducting MVMR were combinations of IVs for each exposure. We restricted the analysis to SNPs that were clumped on r^2^ < 0.001 within 10000 kb.

### Statistical analysis

The presentation of odds ratios (OR) and 95% confidence intervals (95% CI) in the statistical results indicates significance when *P* < 0.05. In order to interpret causality more rigorously, the significance threshold was adjusted according to the false discovery rate (FDR) method. The analyses were conducted using the R 3.5.3 software, which can be found at https://www.Rproject.org. The packages “fdrtool”, “TwoSampleMR” [31] and “MendelianRandomization” in R were utilized for MR analyses and clumping. Finally, MR-PRESSO was performed by the MR-PRESSO package.

## Results

### Causal effect between immunophenotypes and IPF risk

A total of 14,997 SNPs were utilized to analyze 731 immunophenotypes, following the selection criteria for IVs. Supplementary Table 1 displays information regarding the chosen IVs. A two-sample MR analysis was conducted to examine the causal effects of IPF on immunophenotypes, with the IVW method employed as the primary approach. After applying FDR adjustment (P_FDR_ < 0.05), a total of 20 immunophenotypes were identified to have significant associations with IPF (Supplementary Table 2). A total of nine were observed in the TBNK panel, while three were observed in the Treg panel. Additionally, two were observed in each of the myeloid cell, monocyte, and B-cell panels. Furthermore, one was observed in both the myeloid cell and monocyte panels, and one was observed in the T-cell panel at the maturation stage.

**Fig. 2:**
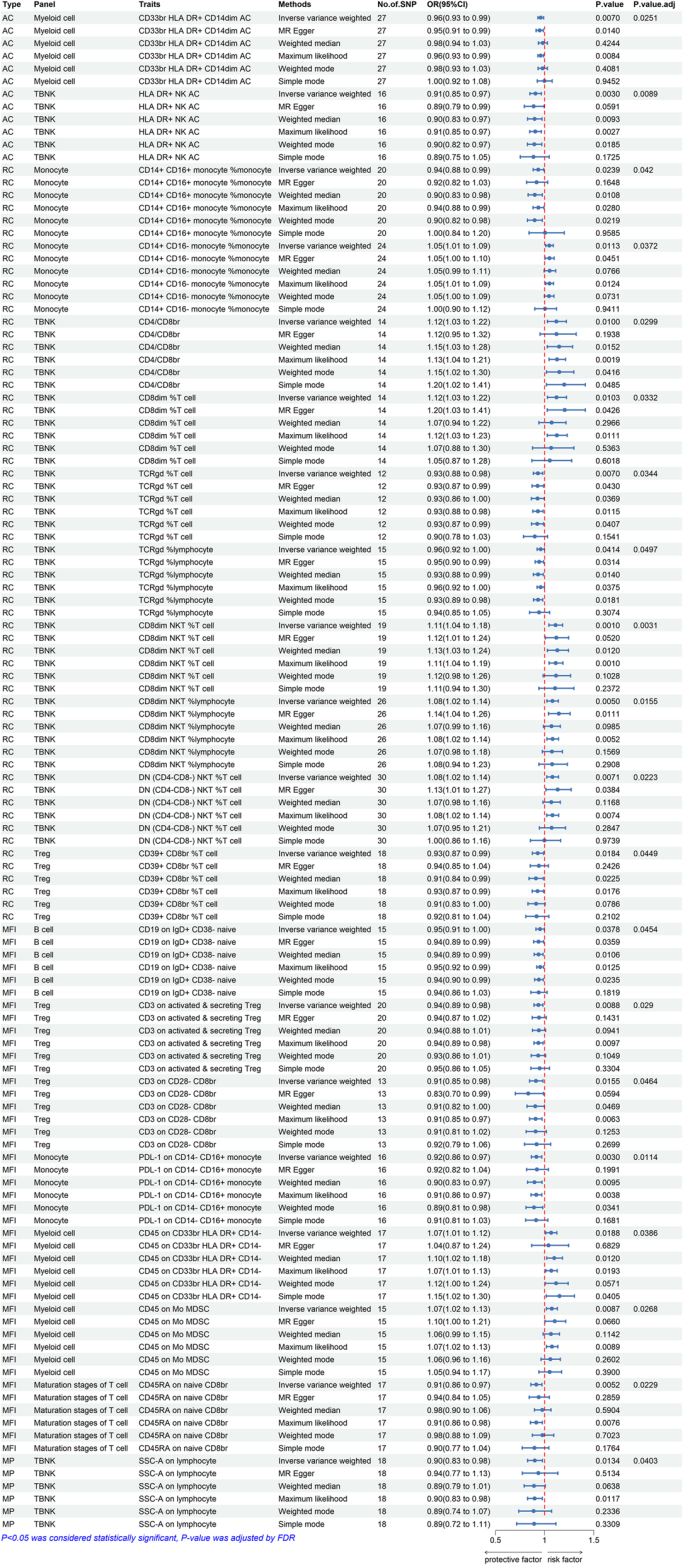
Various techniques were employed in the forest plots to demonstrate the causal connections between immune cell characteristics and IPF.SNP refers to single nucleotide polymorphism, OR stands for odds ratio, and CI represents confidence interval

**Fig. 3:**
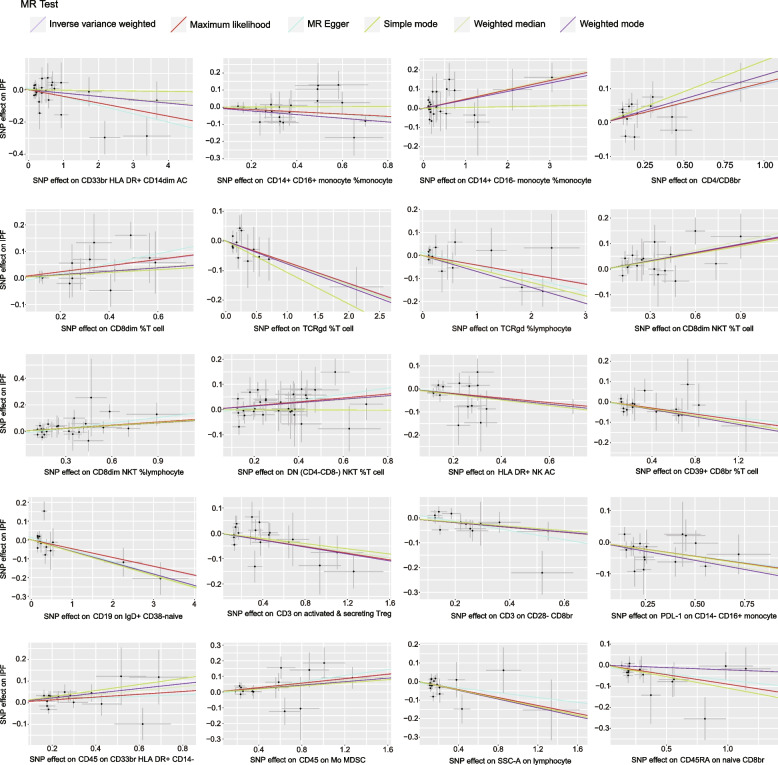
Graphs illustrating the correlation between IPF risk and 20 immune cells. The scatter plot includes an x-axis to illustrate the magnitude of the exposure SNP effect, while a y-axis is utilized to represent the outcome SNP effect

**Fig. 4:**
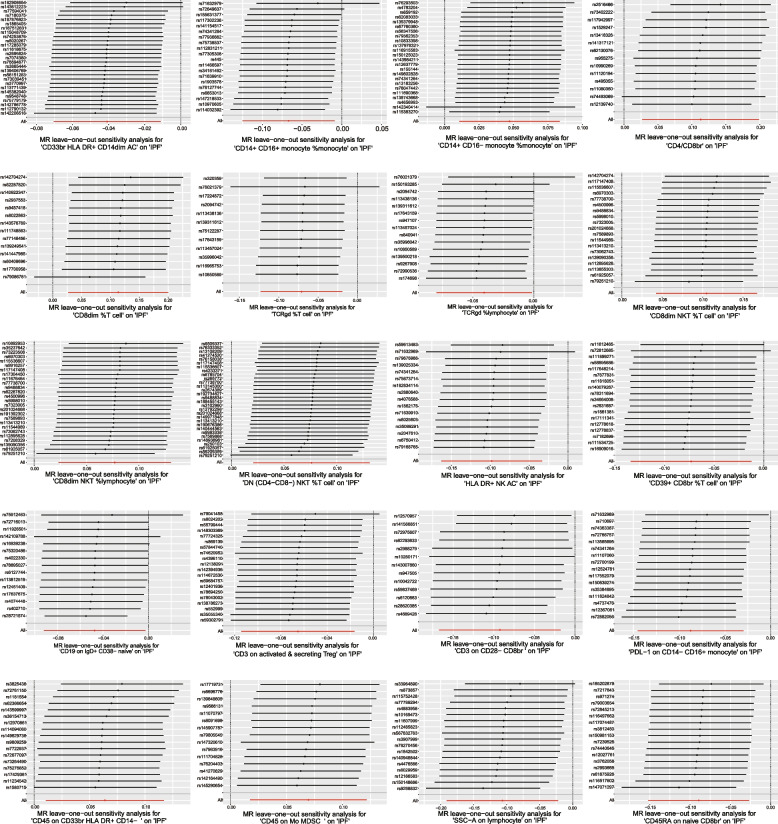
A leave-one-out analysis of 20 immune cells and IPF

Among the 20 immunophenotypes, those positively associated with IPF risk are CD4/CD8br (OR = 1.12, 95% CI 1.03 ~ 1.22, *P* = 0.009, P_FDR_ = 0.029), CD8dim %T cell (OR = 1.12, 95% CI 1.03 ~ 1.22, *P* = 0.010, P_FDR_ = 0.033), CD8dim NKT %T cell (OR = 1.11, 95% CI 1.05 ~ 1.18, *P* = 9.92 × 10^–4^, P_FDR_ = 0.003), CD8dim NKT %lymphocyte (OR = 1.08, 95% CI 1.02 ~ 1.14, *P* = 0.005, P_FDR_ = 0.015), DN (CD4-CD8-) NKT %T cell (OR = 1.08, 95% CI 1.02 ~ 1.14, *P* = 0.007, P_FDR_ = 0.022), CD45 on CD33br HLA DR + CD14- (OR = 1.07, 95% CI 1.01 ~ 1.12, *P* = 0.018, P_FDR_ = 0.038), CD45 on Mo MDSC (OR = 1.07, 95% CI 1.02 ~ 1.13, *P* = 0.008, P_FDR_ = 0.026) and CD14 + CD16- monocyte %monocyte (OR = 1.05, 95% CI 1.01 ~ 1.09, *P* = 0.011, P_FDR_ = 0.037). Of these 20 immunophenotypes, the ones that are negatively correlated with IPF risk are TCRgd %T cell (OR = 0.93, 95% CI 0.88 ~ 0.98, *P* = 0.007, P_FDR_ = 0.034), TCRgd %lymphocyte (OR = 0.96, 95% CI 0.96 ~ 1.00, *P* = 0.041, P_FDR_ = 0.049), HLA DR + NK (OR = 0.91, 95% CI 0.85 ~ 0.97, *P* = 0.002, P_FDR_ = 0.008), SSC-A on lymphocyte (OR = 0.90, 95% CI 0.83 ~ 0.98, *P* = 0.013, P_FDR_ = 0.040), SSC-A on lymphocyte (OR = 0.96, 95% CI 0.93 ~ 0.99, *P* = 0.007, P_FDR_ = 0.025), CD14 + CD16 + monocyte %monocyte (OR = 0.94, 95% CI 0.88 ~ 0.99, *P* = 0.023, P_FDR_ = 0.042), PDL-1 on CD14- CD16 + monocyte (OR = 0.92, 95% CI 0.86 ~ 0.97, *P* = 0.002, P_FDR_ = 0.011), CD39 + CD8br %T cell (OR = 0.93, 95% CI 0.87 ~ 0.99, *P* = 0.018, P_FDR_ = 0.044), CD3 on activated & secreting Treg (OR = 0.94, 95% CI 0.89 ~ 0.98, *P* = 0.008, P_FDR_ = 0.029), CD3 on CD28- CD8br (OR = 0.91, 95% CI 0.85 ~ 0.98, *P* = 0.015, P_FDR_ = 0.046), CD19 on IgD + CD38- naive (OR = 0.95, 95% CI 0.91 ~ 1.00, *P* = 0.037, P_FDR_ = 0.045) and CD45RA on naive CD8br (OR = 0.91, 95% CI 0.86 ~ 0.97, *P* = 0.005, P_FDR_ = 0.022). The results of MR-Egger, weighted median, ML, weighted mode, and simple mode all align with the IVW results. The forest plot of all significant associations is shown as Fig. 2. Furthermore, the presence of horizontal pleiotropy was excluded by both the MR-Egger intercept and the MR-PRESSO global test, as shown in Supplementary Tables 3 and 4. According to the sensitivity analysis, the observed causal associations were robust. The stability of the results was also demonstrated by scatter plots and leave-one-out plots (Figs. 3 and 4). Finally, to evaluate reverse causal effects, we utilized IPF as the exposure, 20 immunophenotypes as the outcome, and 10 SNPs associated with IPF extracted from a previous GWAS as the IVs. Notably, no reverse causality was observed (P_FDR_ > 0.05) (Supplementary Table 5).

### Multivariable MR analysis

**Fig. 5:**
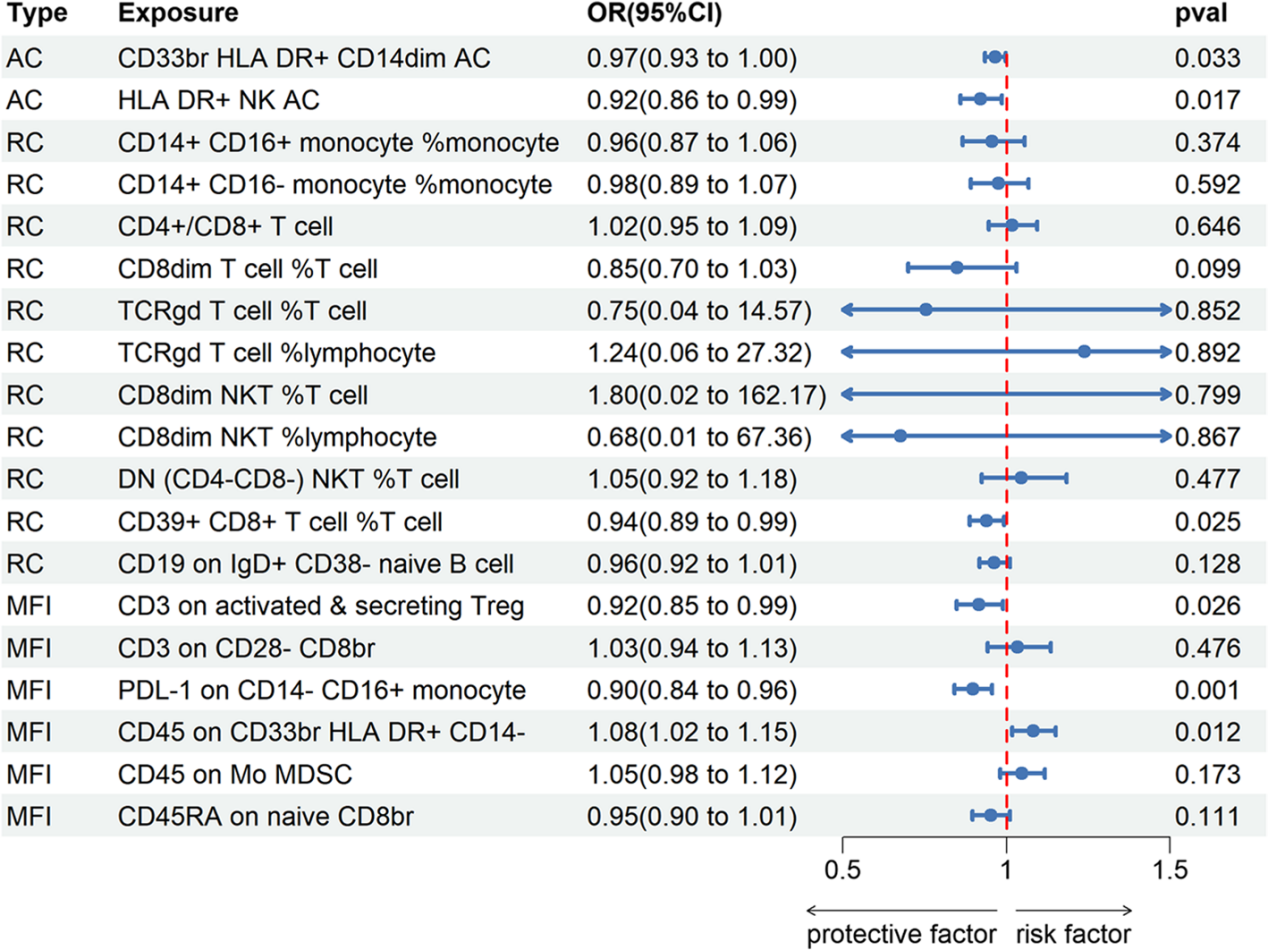
A forest diagram illustrating the association between twenty different immune cells and the risk of developing IPF using multivariable MR analysis.OR, odds ratio; CI, confdence interval

Multivariate MR analysis performed in subgroups based on four types (AC, RC, MP, MFI) of immune traits. The MP type was excluded from the further analysis due to only one immune phenotype. After adjusting for other immune cell characteristics within subgroups, there was a significant causal association between IPF and 6 immune cell traits (Fig. 5): CD33 + HLA DR + CD14dim (OR = 0.96, 95% CI 0.93 ~ 0.99, *P* = 0.033), HLA DR + NK (OR = 0.92, 95% CI 0.85 ~ 0.98, *P* = 0.017), CD39 + CD8 + T cell %T cell (OR = 0.93, 95% CI 0.88 ~ 0.99, *P* = 0.024), CD3 on activated & secreting Treg (OR = 0.91, 95% CI 0.84 ~ 0.98, *P* = 0.026), PDL-1 on CD14- CD16 + monocyte (OR = 0.89, 95% CI 0.84 ~ 0.95, *P* = 8 × 10–4), CD45 on CD33 + HLA DR + CD14- (OR = 1.08, 95% CI 1.01 ~ 1.15, *P* = 0.011). In addition, 13 other immune cell phenotypes (i.e., CD14 + CD16 + monocyte %monocyte, CD14 + CD16- monocyte %monocyte, CD4/CD8br, CD8dim %T cell, TCRgd %T cell, TCRgd %lymphocyte, CD8dim NKT %T cell, CD8dim NKT %lymphocyte, DN (CD4-CD8-) NKT %T cell, CD19 on IgD + CD38- naive, CD45 on Mo MDSC, CD3 on CD28- CD8br, CD45RA on naive CD8br) lost statistical significance after adjustment.

## Discussion

A thorough examination of extensive genetic data was undertaken to explore the genetic association between IPF and 731 immune cell traits. The outcomes of this study provide compelling evidence suggesting the involvement of immune cells in influencing the susceptibility to IPF. The study utilized a two-sample MR method to validate that 20 immune traits were significantly linked to the risk of IPF (P_FDR_ < 0.05). The development of IPF may potentially be linked to various immune cells.

Recently, the results of the CAMPUCITY and INSPIRE trials found a strong association between monocytes and the risk of disease progression and death in IPF, and proposed monocyte counts as a biomarker for predicting prognosis in IPF [32]. The results of our study revealed a negative association between the expression of PDL-1 on CD14- CD16 + monocytes and the susceptibility to IPF. In the innate immune system, monocytes and macrophages serve as immune sentinels, primarily characterized by the presence of CD14 and CD16, which are essential for cellular participation in fibrosis. Phenotypic markers can be utilized to classify monocyte subsets into intermediate (CD16 + CD14 +), classic (CD16-CD14 +) and non-classical (CD16 + CD14 −) categories [33]. Many researches have revealed that CD14 + CD16- classical monocytes can mature into macrophages in the early stages of fibrosis and are closely associated with the development of IPF [33–35]. Interestingly, in our study, IPF declined with increasing CD14 + CD16 + monocyte %monocyte ratio, whereas past studies have indicated that intermediate monocytes are associated with a worse prognosis in IPF patients [36]. Furthermore, it has been reported that the combination of pirfenidone and a PDL-1 inhibitor improves the efficacy of treatment in lung cancer patients with IPF [37]. Blocking the PD-1 pathway also reduces pro-fibrotic factors such as IL-17 and TGF-β production [38]. Thus, these findings provide new insights for us to continue exploring the role of monocytes in IPF and new therapeutic targets.

It has also been found that HLA DR + NK AC is strongly linked to decreased susceptibility to IPF. In various fibrotic model systems, NK cells primarily demonstrate anti-fibrotic properties [39]. In addition, patients with pulmonary infection with mycobacterium tuberculosis have a higher probability of developing IPF, and the number of NK cells in lymphocytes isolated from bronchoalveolar lavage fluid is lower in IPF patients [40]. Currently, pre-activated HLA-DR NK cells in vitro have been considered as a target subpopulation for anti-tuberculosis therapies due to the important role they can play in different stages of mycobacterium tuberculosis infection [41, 42]. Additionally, this phenomenon offers a significant opportunity for impeding the progression of IPF. Moreover, HLA-DR NK cells possess the capacity to selectively bind particular antigens, subsequently presenting them to CD4 and CD8 T cells, thereby inducing their activation and subsequent proliferation [43]. Our study found a negative correlation between the risk of IPF and the percentage of CD39 + CD8 + T cells. The involvement of T cells in IPF remains a subject of debate, with animal models of pulmonary fibrosis showing that T cells can have either harmful or beneficial effects depending on their characteristics. The use of targeted T cells has become less popular due to the ineffectiveness of anti-inflammatory therapies in IPF patients.

Our findings also indicate that an increase in the ratio of CD3 on activated & secreting Tregs could reduce the occurrence of IPF, which is consistent with the previous discovery that Tregs are protective in IPF through reducing fibroblast accumulation and inhibiting inflammatory responses [44, 45]. Recent data suggest that Tregs might play diverse roles in the early and late stages of fibrosis. In addition, cells like CD8dim NKT %T cell and CD8dim NKT %lymphocyte can increase the risk of IPF. CD8dim NKT belongs to the classical NKT cells with dual immunoregulatory roles and is able to secrete pro-fibrotic factors such as IFN-γ and IL-17A [46]. IL-17A, in particular, can trigger epithelial-mesenchymal transition (EMT) in alveolar epithelial cells through TGF-β. This process is believed to contribute to lung fibrosis. By blocking IL-17A through TGF-β1-dependent and non-dependent mechanisms, it is possible to suppress lung inflammation and fibrosis [47, 48]. Current research on the role of immune cells in IPF remains limited. In addition to the immune cells mentioned above, there are also some cells such as TCRgd% T cells that have been shown in previous experimental reports to produce IFN-γ, thereby inhibiting the progression of IPF. However, there are numerous immune phenotypes that have not yet been thoroughly investigated by researchers in our study and the underlying mechanisms remain largely unknown. In addition, studies of various immunophenotypes are commonly used by investigators to predict and determine prognosis. For example, T-cell related proteins and genes, LCK and CD28, have been used as prognostic biomarkers for IPF [49]. In terms of clinical treatments, the recently proposed mesenchymal stem cell(MSC) therapy is continuously supported by many preclinical evidences. The mouse model of this therapy showed a significant reduction in inflammatory cells and even an increase in regulatory T cells with anti-inflammatory effects, making this a promising therapeutic strategy [50]. With the exception of pirfenidone and nintedanib, most of the drugs are still in clinical trials. In the latest study [51], TH5487 targeted inhibition of OGG1, inflammatory macrophages and neutrophils were found to be significantly reduced and ameliorated lung injury, so this may lead to a new way of thinking about designing the appropriate drug to treat IPF.

The current study is grounded on a two-sample MR analysis, utilizing published findings from a substantial GWAS cohort comprising approximately 150,000 individuals, thereby ensuring its statistical robustness. The study's findings were derived from genetic IVs, and causal inferences were drawn using various MR analysis techniques. Furthermore, our results exhibit robustness, as they remain unaffected by horizontal pleiotropy and other confounding factors.

The limitations of this study must, however, be acknowledged. Firstly, despite implementing the FDR multiple correction, the liberal threshold for SNP selection owing to the constrained sample size may lead to a certain extent of spurious positive findings. Subsequently, despite conducting multiple sensitivity analyses, a comprehensive evaluation of horizontal pleiotropy could not be achieved. Thirdly, our analyses were conducted using aggregated-level datasets, with individual-level data being unavailable. Consequently, the present study was unable to perform additional stratification of the traits of interest based on group characteristics such as gender, age, etc. Last, since this research relies on a European database, the outcomes cannot be extrapolated to other ethnicities, thereby restricting the generalizability of our findings.

## Conclusion

To summarize, our results indicate that 20 immune cells may play a causal role in IPF based on a thorough bidirectional MR analysis. In our study, we have successfully mitigated the effect of inevitable confounding variables, reverse causality, and additional factors that might influence our findings. Consequently, it offers a promising avenue for researchers to investigate the fundamental biological mechanisms of IPF, gain novel insights into the immunology of IPF pathogenesis, and acquire valuable indications for early intervention and treatment.

### Supplementary Information

**Supplementary Material 1.**

**Supplementary Material 2.**

**Supplementary Material 3.**

**Supplementary Material 4.**

**Supplementary Material 5.**

## Data Availability

The datasets presented in this study can be found in online repositories. Data URLs: GWAS summary statistics for 731 immune traits could be download form GWAS Catalog (Study accession: GCST90001001—GCST90002000, https://www.ebi.ac.uk/gwas/home); IPF could be available form http://results.globalbiobankmeta.org/pheno/IPF. All codes used in the research are available from the corresponding authors.

## Abbreviations

AC: Absolute cell
CI: Confidence interval
EMT: Epithelial-mesenchymal transition
FDR: False discovery rate
GWAS: Genome-wide association study
GBMI: Global Biobank Meta-analysis Initiative
HLA: Human leukocyte antigen
IPF: Idiopathic pulmonary fibrosis
IL: Interleukin
IVs: Instrumental variables
IVW: Inverse variance weighting
LD: Linkage disequilibrium
MR: Mendelian Randomization
MFI: Median fluorescence intensities
MP: Morphological parameters
ML: Maximum likelihood
MR-PRESSO: MR pleiotropy residual sum and outlier
MVMR: Multivariate mendelian randomization
NK: Natural killer
OR: Odds ratio
RC: Relative cell
SNPs: Single nucleotide polymorphisms
Tregs: Regulatory T cells
